# Supplementation With Bifidobacterium and Symptomatic Control in Irritable Bowel Syndrome: An Evidence-Based Review

**DOI:** 10.7759/cureus.44447

**Published:** 2023-08-31

**Authors:** Inês Terra, Catarina Santeiro, Jenifer da Fonseca Rua

**Affiliations:** 1 Family Medicine, Unidades de Saúde Familiar (USF) Casa dos Pescadores, Póvoa de Varzim, PRT; 2 Family Medicine, Unidades de Saúde Familiar (USF) Lusitânia, Évora, PRT; 3 Family Medicine, Unidades de Saúde Familiar (USF) Conde de Oeiras, Oeiras, PRT

**Keywords:** irritable bowel syndrome, abdominal pain, probiotics, symptomatic control, bifidobacterium

## Abstract

Irritable bowel syndrome (IBS) is a functional gastrointestinal disorder, with a global prevalence of around 11%. Family doctors should be aware of the diagnosis and treatment of this pathology. The benefit of using probiotics is questionable. The purpose of this review is to establish the evidence of the association between *Bifidobacterium* supplementation and symptomatic control in patients with IBS. The research was conducted using the National Guideline Clearinghouse, National Electronic Library for Health of the British NHS, Canadian Medical Association Practice Guidelines InfoBase, Cochrane Library, Database of Abstracts of Reviews of Effectiveness, Bandolier, Evidence-Based Medicine Online, and PubMed. Articles published between March 2017 and March 2022 in humans and written in Portuguese, Spanish, and English using the terms IBS and *Bifidobacterium *were included*.* To stratify the level of evidence (LOE), the Strength of Recommendation Taxonomy (SORT), from the American Academy of Family Physicians, was used. Thirty-seven articles were found corresponding to the search terms, and a total of seven articles were selected. Three clinical trials and a simple review have demonstrated improvement in symptoms, although further studies are needed. The guideline and the systematic review did not demonstrate superiority in symptomatic relief when compared to other species of probiotics. The meta-analysis did not show the efficacy of the isolated use of *Bifidobacterium.* The evidence of an association between supplementation with *Bifidobacterium* and symptomatic control in patients with IBS is not clear. Some studies seem to demonstrate benefits in improving symptoms (SORT C).

## Introduction and background

Irritable bowel syndrome (IBS) is a group of symptoms that occur together including abdominal pain and alterations in bowel habits, without any organic disease [1,2]. The Rome IV criteria included recurrent abdominal pain associated with defecation and changes in frequency and/or stool consistency [3-5]. It is a chronic condition that can have an impact on the quality of life, being associated with high rates of absenteeism and a significant portion of healthcare costs [1,4,5]. The estimated prevalence of IBS is 11% worldwide, and the pathophysiology is multifactorial, poorly understood, and influenced by a disturbance in the brain-gut axis [1,5-8].

Family physicians are usually the first point of contact and the ones who make a positive diagnosis of IBS after excluding the alarm signs that warrant referral [6,9]. Establishing a trusting doctor-patient relationship and shared decision-making is the key to the accurate diagnosis and proper management of chronic diseases such as IBS [10]. Therapeutically, although there are medications that can improve symptoms, there is no cure for this condition. Probiotics may influence IBS symptoms, including abdominal pain, bloating, flatulence, altered bowel movements, and intestinal microbiota [10-12]. Studies conducted so far have used different species, doses, and formulations, so their benefit remains controversial [10-14]. The aim of this review is to explore the evidence regarding the association between supplementation with *Bifidobacterium* and symptomatic control among patients with IBS.

## Review

Methods

Research was conducted on clinical trials, guidelines, meta-analyses, systematic reviews, and narrative reviews, using the Medical Subject Heading (MeSH) terms “irritable bowel syndrome” and “​​​​*Bifidobacterium*,” in the following databases: National Guideline Clearinghouse, National Electronic Library for Health of the British NHS, Canadian Medical Association Practice Guidelines InfoBase, Cochrane Library, Database of Abstracts of Reviews of Effectiveness, Bandolier, Evidence-Based Medicine Online, and PubMed.

We included articles published between March 2017 and March 2022 in humans and written in Portuguese, English, and Spanish languages that met the following eligibility criteria: population, adult individuals with IBS; intervention, treatment with *Bifidobacterium*; comparison, placebo/no treatment with *Bifidobacterium*; outcome, the effect of *Bifidobacterium* supplementation and symptomatic control among patients with IBS (PICO).

**Figure 1:**
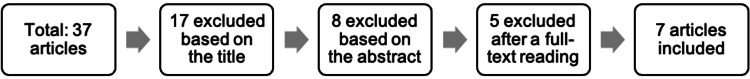
Selection of the articles

Duplicate articles, articles that did not meet the PICO criteria, and articles with non-inclusive typologies for this study were excluded. The selection of articles based on title and abstract was independently performed by the three authors (Figure 1). Articles selected for full-text reading were reviewed by at least two of the authors to decide their inclusion in case of doubt. The final evaluation of the quality and level of evidence (LOE) of the included articles was discussed and decided by consensus among all authors. To assess the levels of evidence (LOE) and assign grades of recommendation strength, the Strength of Recommendation Taxonomy (SORT) scale from the American Academy of Family Physicians was applied.

Results

In the following table (Table 1), we analyze the clinical trials included in this review, which do not allow definitive conclusions to be drawn about the effectiveness of the use of supplementation. The clinical trials included in this review are listed in Table 1, and for each of them, we synthesized the methods, results, and limitations.

**Table 1:** Clinical trials LOE: (A) Strong research-based evidence (multiple, relevant, high-quality studies with homogeneous results, e.g., two or more randomized controlled trials or a systematic review with clearly positive results) and (B) moderate evidence (e.g., one randomized controlled trial or multiple adequate studies) LOE, level of evidence; IBS, irritable bowel syndrome

References	Methodology	Results	Limitations	LOE
Clinical trials				
“Heat-inactivated *Bifidobacterium bifidum* MIMBb75 (SYN-HI-001) in the treatment of irritable bowel syndrome: a multicentre, randomised, double-blind, placebo-controlled clinical trial” [13]	Group A: two placebo capsules; group B: two capsules with heat-inactivated *B. bifidum* MIMBb75 cells. Oral administration, daily, eight weeks. Germany	Improvement in abdominal pain: 34% in group B compared to 19% in group A	Significant response to placebo. The use of Rome III criteria	A
“*Lactobacillus acidophilus* DDS-1 and *Bifidobacterium lactis* UABla-12 Improve Abdominal Pain Severity and Symptomology in Irritable Bowel Syndrome: Randomized Controlled Trial” [8]	Group 1: placebo; group 2: *L. acidophilus*; group 3: *B. animalis*. Oral administration, daily, six weeks. India	Change in severity of abdominal pain in both groups, compared to placebo with 52.3% in group 2 and 28.2% in group 3	Unique composition of the Indian gut microbiome	A
“Efficacy of *Lactobacillus paracasei* HA-196 and *Bifidobacterium longum* R0175 in Alleviating Symptoms of Irritable Bowel Syndrome (IBS): A Randomized, Placebo-Controlled Study”[1]	Group 1: placebo; group 2: *L. paracasei*; group 3: *B. longum*. Oral administration, daily, eight weeks. Canada	Improvement in the severity of symptoms in all groups. No significant improvement in IBS-constipation group. In the IBS-diarrhea group, significant improvement in stool frequency. Improvement in mental health in group 3	Response to placebo. Higher number of females compared to males in the group in question. The use of Rome III criteria	B

The meta-analysis “Efficacy of *Bifidobacterium infantis *35624 in patients with irritable bowel syndrome: a meta-analysis” [2] that reviews randomized clinical trials, in which the use of *B. infantis* was compared with placebo, selected five studies, three of which were exclusively on *B. infantile *666, and concluded that treatment with only *B. infantis *666 did not affect the symptoms, and its effectiveness was not confirmed. LOE was A, which is strong research-based evidence (multiple, relevant, high-quality studies with homogeneous results, e.g., two or more randomized controlled trials or a systematic review with clearly positive results).

The systematic review included in the study “The Effect of *Bifidobacterium* on Reducing Symptomatic Abdominal Pain in Patients with Irritable Bowel Syndrome: A Systematic Review” [14] compared probiotic to placebo, and the primary outcome was the improvement of abdominal pain. Eight studies were selected, approximately 1045 participants. Not all studies showed significant improvement in abdominal pain; no participants reported increased abdominal pain or other adverse effects. The limitation of this study was the heterogeneity of the effect of *Bifidobacterium *depending on the species, dosage, and formulation. LOE was B, which is moderate evidence (e.g., one randomized controlled trial or multiple adequate studies).

The guideline included in the review “British Society of Gastroenterology guidelines on the management of irritable bowel syndrome” [9] concludes that probiotics may be effective in relieving global symptoms, particularly abdominal pain; however, it is not possible to recommend a specific species. It is reasonable to advise patients who wish to try probiotics to take them for a period of up to 12 weeks. LOE was C, which is limited research-based evidence (e.g., controlled prospective studies).

The narrative review “*Bifidobacteria* and Their Health-Promoting Effects” [12] concluded that *B. animalis *HN019 has shown to improve gastrointestinal function, while *B. animalis *DN171010 reduced IBS symptoms. *Bifidobacterium infantis* has demonstrated the ability to reduce pro-inflammatory cytokines, not only at the gastrointestinal level but also systemically. Further studies with robust scientific evidence are needed to support the beneficial effects of this particular group of gut bacteria. LOE was C, which is limited research-based evidence (e.g., controlled prospective studies).

## Conclusions

The evidence of an association between supplementation with *Bifidobacterium* and symptomatic control in patients with IBS is not clear. Some studies seem to demonstrate benefits in improving symptoms (SORT C).

Despite the heterogeneity of the results found, supplementation with *Bifidobacterium* appears to be useful in treating IBS symptoms and has not been associated with significant adverse effects. Still, it is not possible to recommend any specific probiotic; however, there is some benefit in treating general symptoms and abdominal pain. It is necessary to evaluate the anti-inflammatory effect of *Bifidobacterium* strains in patients with IBS. This review was useful in stratifying the available evidence, which is currently still limited and heterogeneous, hence the need for more studies.
